# Interaction between Bovine Serum Albumin in Fresh Milk Cream and Encapsulated and Non-Encapsulated Polyphenols of Tamarillo

**DOI:** 10.3390/antiox12081611

**Published:** 2023-08-14

**Authors:** Chen Liu, Bao Viet Nguyen, Tung Thanh Diep, Michelle Ji Yeon Yoo

**Affiliations:** 1 School of Science, Faculty of Health and Environment Sciences, Auckland University of Technology, Private Bag 92006, Auckland 1142, New Zealand; 2 Department of Chemical Engineering, Nong Lam University, Ho Chi Minh City 70000, Vietnam; nbviet@hcmuaf.edu.vn (B.V.N.); dttung@hcmuaf.edu.vn (T.T.D.)

**Keywords:** polyphenol interaction, BSA, cream, chlorogenic acid, kaempferol-3-rutinoside, delphinidin-3-rutinoside, pelargonidin-3-rutinoside, encapsulated polyphenols

## Abstract

The fortification of dairy products with polyphenols is known to deliver additional health benefits. However, interactions between polyphenols may form complexes and cause a loss of functionality overall. This study aimed to investigate potential interactions between polyphenols, in encapsulated and non-encapsulated forms, extracted from tamarillo fruit and bovine serum albumin (BSA) from fresh milk cream. Fortification with tamarillo extract was made at 1, 2 and 3% (*w*/*w*), and the resultant changes in physicochemical, rheological and functional properties were studied. With an increase in fortification, the absorbance of protein–ligand in the protein–polyphenol complex was decreased by up to 55% and 67% in UV and fluorescent intensities, respectively. Chlorogenic acid and kaempferol-3-rutinoside were more affected than delphinidin-3-rutinoside and pelargonidin-3-rutinoside. Static quenching was the main mechanism in the fluorescence spectra. Tryptophan and tyrosine residues were the two major aromatic amino acids responsible for the interactions with BSA. There were at least three binding sites near the tryptophan residue on BSA. The rheological property remained unaffected after the addition of non-encapsulated tamarillo extracts. Antioxidant capacity was significantly decreased (*p* < 0.05) after the addition of encapsulated extracts. This may be explained by using a low concentration of maltodextrin (10% *w*/*w*) as an encapsulating agent and its high binding affinity to milk proteins.

## 1. Introduction

Tamarillo fruit (*Solanum betaceum*), also known as tree tomato, is a good source of bioactive compounds, containing different polyphenols and anthocyanins [1]. Delphinidin-3-rutinoside, pelargonidin-3-rutinoside, chlorogenic acid and kaempferol-3-rutinoside are the four most abundant polyphenols in the pulp of Laird’s Large (red) cultivar, present at 254.76, 200.66, 66.35 and 50.04 mg in 100 g of freeze-dried powder, respectively [1]. They are present in sufficient amounts per tamarillo fruit to provide antioxidant and anti-cancer properties. For example, daily consumption of up to 10 mg of kaempferol-3-rutinoside reduces the risk of cardiovascular diseases and breast and pancreatic cancers [2]. Although polyphenols and anthocyanins are present in large quantities in fresh fruit, they are easily lost during food processing due to their sensitivity to light, temperature and auto-oxidation [3]. Microencapsulation can be applied to protect bioactive compounds, including polyphenols, from degradation. Both spray-drying and freeze-drying methods are widely used; however, freeze-drying is more effective to prevent bioactive compounds from oxidation because it uses low temperatures. When used with ultrasonication to homogenize the microcapsule particles of polyphenol with maltodextrin to form a complex, the compounds can be further protected to maintain their functionality [4]. It is also important to choose an appropriate encapsulating agent. Out of all the polysaccharides, proteins and lipids that are commonly used as encapsulating agents, maltodextrin is an attractive encapsulating agent due to its low molecular weight, high water solubility and low viscosity [5]. Such characteristics enable easier application of the encapsulated bioactive compounds to foods with fortification.

Research on dairy products and polyphenols has been expedited in the past couple of decades. Studies on interactions between milk proteins, such as casein micelles, whey proteins and milk fat globule membrane proteins, and polyphenols, have been extensively reported in the literature. Such interactions are important to note as the health benefits and functionality of polyphenols could be lost with the formation of new complexes between them. Interactions between polyphenols from tea and cocoa powder with casein, whey proteins and lactoglobulins (α and β conformational globulins) based on the composition of fat content can be found in [6,7]. In comparison with other milk proteins, bovine serum albumin (BSA) is not the dominant protein present in dairy products, but it can be considered a replacement for human serum albumin with a similar structure. Because the interaction between BSA and polyphenols may also occur between human serum albumin and polyphenols, a deeper understanding of the interactions between BSA and polyphenols may help in developing functional dairy foods fortified with polyphenols. Interactions between polyphenols, such as caffeoylquinic acid in cocoa and tea, and BSA using multiple spectroscopic methods have been reported [8]. However, most studies have focused on the molecular dockings between amino acids in milk proteins rather than examining the resultant changes in the physicochemical properties of dairy products.

This study, therefore, focused on the technical and nutritional aspects of cream fortified with encapsulated/non-encapsulated tamarillo extract. We not only investigated potential interactions between milk proteins and major polyphenol compounds (chlorogenic acid, delphinidin-3-rutinoside, pelargonidin-3-rutinoside and kaempferol-3-rutinoside) of tamarillo fruit but also evaluated the effects of tamarillo addition on physicochemical properties (rheology, phenolic content, anthocyanin content, and antioxidant capacity) of this dairy product.

## 2. Materials and Methods

### 2.1. Materials

Laird’s Large red tamarillos were collected from growers in the Northland region of New Zealand with commercial maturity of 21–24 weeks from anthesis. Fresh pasteurized milk cream was purchased from a local supermarket (Anchor cream with >37 g fat, 3 g carbohydrates and 2.4 g proteins in 100 mL). All chemicals and reagents, including bovine serum albumin (BSA), maltodextrins (encapsulating agent), 20% Na_2_CO_3_, 0.01 M CuCl_2_, 1.0 M NH_2_Ac buffer with pH at 7.0, 0.0075 M neocuporine and Folin–Ciocalteu phenol reagent were purchased from Sigma-Aldrich (Auckland, New Zealand). Standards of polyphenols, including chlorogenic acid, delphinidin-3-rutinoside, pelargonidin-3-rutinoside and kaempferol-3-rutinoside were obtained from Extrasynthese (Genay Cedex, France). Isopropanol (IPA), 70% acetone, 99.5% formic acid, toluene and 50% methanol were of liquid chromatography-mass spectroscopy (LCMS) grade, and they were obtained from Thermo Fisher (Auckland, New Zealand). Milli-Q water purified with a purifying machine (Purite Limited, Thame, Oxon, UK) was used. Pectinase (Novozyme (Copenhagen, Denmark)) was obtained from Azelis New Zealand.

### 2.2. Extraction of Tamarillo

The extraction process was modified from [9]. Pulps of peeled tamarillos were cut into pieces and acidified water of citric acid (2% *w*/*v*) was added in a ratio of 1:5 (pulp: acidified water, *w*/*w*) and ground in a blender. The extracted solution was collected in a Schott bottle followed by the addition of pectinase (Novozyme), according to the manufacturer’s instructions. The purpose of adding pectinase was to decompose plant cells for better extraction. The extracts of tamarillo were centrifuged at 10,000× *g* for 10 min, and the aqueous supernatant was collected into 20 mL centrifuge tubes and stored in a freezer at −80 °C. Then, these samples were placed in a freeze-drier (Alpha 1–2 LD plus Freeze Dryer, Martin Christ, New Zealand) with the application of a high-pressure vacuum at −80 °C for 4 days and then collected in a zipped plastic bag and stored at −20 °C for further experiments.

#### 2.2.1. Encapsulation of Extracted Tamarillo

The tamarillo extract (Section 2.2) with a pH of 4.0 ± 0.5 was homogenized and encapsulated with maltodextrin (10%, *w*/*w*) using an ultrasonic homogenizer (Bueno Biotech Co., Ltd., BEM-150A, Nanjing, China) under 80% power with probe No. 6 for 7 min at ambient temperature. The dehydration was conducted using a freeze-drier (Alpha 1–2 LD plus Freeze Dryer, Martin Christ, New Zealand) at −80 °C for 4 days, and then the encapsulated powders (with a moisture content of 7% [9]) were ground to a size of 1 mm using a mortar grinder. Then, they were packed and stored under −20 °C for further analysis. Refer to Supplementary Figure S1 for a photograph showing the encapsulated extracts.

#### 2.2.2. Preparation of Fresh Milk Cream Samples with Polyphenols

The fresh milk cream was stored in a fridge, which was set at 4 °C for at least 24 h. Freeze-dried tamarillo extract, either encapsulated (Section 2.2.1) or non-encapsulated (Section 2.2), was added to the cream at 1%, 2% or 3% *w*/*w*. The milk cream–polyphenol complex was homogenized with a magnetic stirring bar at low speed to prevent it from foaming. The samples were further tested for rheological property, particle size, total phenolic contents and antioxidant capacity.

### 2.3. Preparation of Standard Solutions

The stock solution of BSA was prepared with Milli Q pure water at room temperature with a concentration of 1 × 10^−5^ M. The stock solution of chlorogenic acid, kaempferol-3-rutinoside, delphinidin-3-rutinoside and pelargonidin-3-rutinoside was dissolved with methanol to a concentration of 1.0, 2.0, 10.0 and 5.0 g.L^−1^, respectively.

### 2.4. Spectroscopic Analysis

#### 2.4.1. UV-Visible Absorption Spectroscopy

Following the methods of [8], the stock solution of BSA was kept at a concentration of 1 × 10^−5^ M. The UV-visible absorption spectra of BSA in the presence and the absence of chlorogenic acid, kaempferol-3-rutinoside, delphinidin-3-rutinoside and pelargonidin-3-rutinoside, were measured using a UV-spectrophotometer (Ultrospec 7000, Cambridge, UK) in a range of 190–350 nm at room temperature. The concentration of polyphenols was kept the same as BSA, i.e., at 1 × 10^−5^ M.

#### 2.4.2. Fluorescence and Synchronous Fluorescence Spectroscopy

The binding site and binding constant were determined using fluorescence spectra with modifications from [8]. The concentration of BSA was set at 1 × 10^−6^ M. The concentrations of chlorogenic acid, kaempferol-3-rutinoside, delphinidin-3-rutinoside and pelargonidin-3-rutinoside were set at 0, 1.0, 2.0, 3.0, 4.0, 5.0, 6.0, 7.0 and 8.0 × 10^−5^ M, individually. The fluorescence spectra of the BSA–polyphenol complexes were recorded using a Cary Eclipse fluorescence spectrophotometer (MY17010001, Agilent, Santa Clara, CA, USA) at room temperature with an excitation wavelength set at 280 nm. The conformational change in BSA was recorded from the emission wavelength, with a range of 300 to 500 nm. The binding parameters for the quenching process were calculated using the intensity of the maximum emission wavelength. The emission wavelength of the BSA–polyphenol mixture for synchronous fluorescence spectra was measured between 200 and 500 nm with a Δλ set at 15 and 60 nm. The bandwidth between excitation and emission was kept at 5 nm.

### 2.5. Physicochemical Properties of Fresh Milk Cream Fortified with Polyphenols

#### 2.5.1. Viscosity

The rheological property of the fresh milk cream samples fortified with either encapsulated or non-encapsulated tamarillo extracts was measured using a rheometer (Model 7123075, RST-SST Rheometer) equipped with a spindle RST-CCT-40 (Ametek Brookfield, Middleboro, MA, USA). The cream samples and the spindle cup were kept in a refrigerator at 4 °C for at least 24 h to ensure that the fat molecules were fully crystallized. The total weight of the cream samples including the spindle cup was recorded constant as 252 g. The viscosity was then recorded in 1000 points with a shear rate up to 400 1/S for 120 s. All measurements were made in triplicates.

#### 2.5.2. Particle Size

The particle size of the fortified fresh milk cream samples was measured using a Zetasizer Nano ZS (Malvern Instruments Ltd., Worcestershire, UK). The cream samples were diluted by 10 factors to obtain a clear signal as the dispersity of the sample was too high to measure the particle size. The equipment temperature was set at 20 °C. The unit of particle size was expressed as nm. Z average and *D* [3, 2] were used to determine the particle size of the cream mixture with the equation $D\left[3,2\right]=\frac{\sum_{1}^{n}d^{3}}{\sum_{1}^{n}d^{2}}$, where $D\left[3,2\right]$ refers to the surface moment mean diameter, which is also called the volume to surface mean, $d^{3}$ refers to the volume of a particle, and $d^{2}$ refers to the surface area of the particle [10]. All measurements were made in triplicates.

#### 2.5.3. Extraction and Quantification of Phenolics and Anthocyanins

Phenolics and anthocyanins were quantified using LC-MS/MS consisting of an Agilent 1260 Infinity Quaternary LC System (Santa Clara, CA, USA) and an Agilent 6420 triple quadrupole mass spectrometer with multimode ionization source (model G1978B). For phenolic content, 50 mg of the dried sample (original tamarillo extract) or 0.1 g of the fortified cream sample was placed into a 2 mL screw amber bottle. Then, 1 mL of 50% methanol was added to each bottle. The mixture was vortexed and stored in a fridge for 30 min. The mixture was then centrifuged at 4000 RCF for 5 min at 4 °C. The supernatant was separated and transferred using a glass syringe into a vial insert inside a 1.8 mL amber glass vial and sealed. The final solution was kept at −20 °C for further analysis.

For anthocyanin content, the same weight of samples was added in a 2 mL screw thread amber bottle. Then, 50 µL of 99.5% formic acid, 200 µL of IPA and 400 µL of Milli-Q water were added to this amber bottle. The mixture was vortexed and sonicated at 50 °C for 20 min. Next, 1 mL of toluene was added to the mixture for liquid separation. Then, the mixture was vortexed again and centrifuged at 10,000 RCF for 10 min. The transparent liquid extracts were transferred to a new 60 µL Eppendorf tube. The new tube was centrifuged at 10,000× *g* for 5 min. Then, 50 µL of the lower aqueous phase and 50 µL of Milli-Q water were added to a 0.1 mL glass insert in a 1.5 mL amber bottle and sealed. The final solution was kept at −20 °C for further analysis. The results were reported as µg of polyphenols per ml of the sample solution. All measurements were made in triplicates.

Phenolics were analyzed using liquid chromatography and mass spectrometer with a multimode source. The extracted phenolics were separated in a Cortecs C18 column (2.1 × 100 mm, 2.7 μm). The mobile phase was made of 0.1% formic acid in Milli-Q water (A) and 0.1% acetic acid in acetonitrile (B). The injection volume of samples was 2 μL with a 0.25 mL/min flow rate. Each sample was run using a mass spectrometer in the negative mode for 29 min. The autosampler was set at 4 °C. The multimode source was operated at electrospray ionization parameters with the gas at 337 °C. The capillary voltage was set at 2000 V with a pressure of 60 psi, and the column temperature was set at room temperature.

For anthocyanins, the concentration was determined using fluorescence detection with XSelect C18 column (2.1 × 100 mm, 3.5 μm). The mobile phase of 0.1% formic acid in acetonitrile (A) and 0.6% formic acid in Milli-Q (B) was used. The injection volume of a sample was 2 μL with a run time of 18 min. The mass spectrometer was run in negative mode at electrospray ionization parameters with the gas at 423 °C. The capillary voltage was set at 2000 V with 60 psi.

#### 2.5.4. Total Phenolic Content and Antioxidant Capacity

Total phenolic content was measured using the Folin–Ciocalteu method, which was modified from [1]. The standard solution of gallic acid was made from 0.0039062 to 0.0625 mg.mL^−1^ to produce the standard curve of absorbance against the concentration of gallic acid using UV spectroscopy. The 0.1 g of powder sample or 0.6 g of fortified cream sample were added in a centrifuge tube along with 4 mL of 50% methanol. The mixture was homogenized at room temperature for 1 h and then centrifuged at 1500 RCF for 15 min. The top layer of supernatant was transferred to a 10 mL volumetric flask, and then 4 mL of 70% acetone was added instead of 50% methanol to this centrifuge tube. The homogenization and centrifugation steps were repeated under the same conditions. Then, the volumetric flask was filled up to the 10 mL mark with deionized water. After this, the solution was diluted further by a factor of 10 to produce the sample solution. A 1 mL of sample solution or standard solution and 500 µL of Folin–Ciocalteu phenol reagent were added to a 10 mL brown glass vial and kept at room temperature for 5 min to react. Then, 1.5 mL of 20% Na_2_CO_3_ was added to the mixture. After incubation at room temperature for 2 h while covered in foil, the solution was measured under UV spectra in a cuvette with absorbance at 765 nm against the blank. The results were reported in mg of gallic acid equivalents per 100 g of the extracted polyphenols. All measurements were made in triplicates.

The antioxidant capacity was determined using the CUPRAC method, which was modified from [1]. The standard solution was made of Trolox reagent with a range of concentration from 2.5 mg.L^−1^ to 80 mg.L^−1^ to produce a standard curve of absorbance against the concentration of ascorbic acid using UV spectroscopy. Briefly, 0.1 g of dried tamarillo extracts or 0.6 g of the fortified cream samples were placed in a centrifuge tube along with 4 mL of 50% methanol. The tube was homogenized for 1 hr at room temperature and then centrifuged at 1500 RCF for 15 min. The supernatant from the top was transferred to a 10 mL volumetric flask, and then 4 mL of 70% acetone was added to the tube. The homogenization and centrifugation were completed as described above under the same conditions. Then, the supernatant was transferred into the same volumetric flask that was filled up to the 10 mL mark after the addition of all the supernatant. Then, the solution was diluted into another 10 mL volumetric flask to obtain the final sample solution. A 1 mL of sample solution or standard solution was mixed in a 10 mL brown glass vial with 1 mL of 0.01 M copper chloride, 1 mL of 1.0 M NH_2_Ac buffer at pH 7.0, 1 mL of 0.0075 M neocuporine and 0.1 mL of distilled water. After allowing 5 min for the reaction to occur, the antioxidant capacity of the sample solution was recorded with the wavelength at 450 nm. All measurements were made in triplicates. The results were reported as mg of Trolox equivalents (TEAC) per 100 g of the extracted powder.

### 2.6. Statistical Analysis

All measurements were made in triplicates. The results were reported as mean ± standard deviations. Different groups of data were compared with two-way ANOVAs using R Studio (version 3.4.1) with α at the 0.05 significance level to indicate a significant difference.

## 3. Results

### 3.1. Spectroscopic Analysis

#### 3.1.1. UV-Visible Absorption Spectroscopy

**Figure 1:**
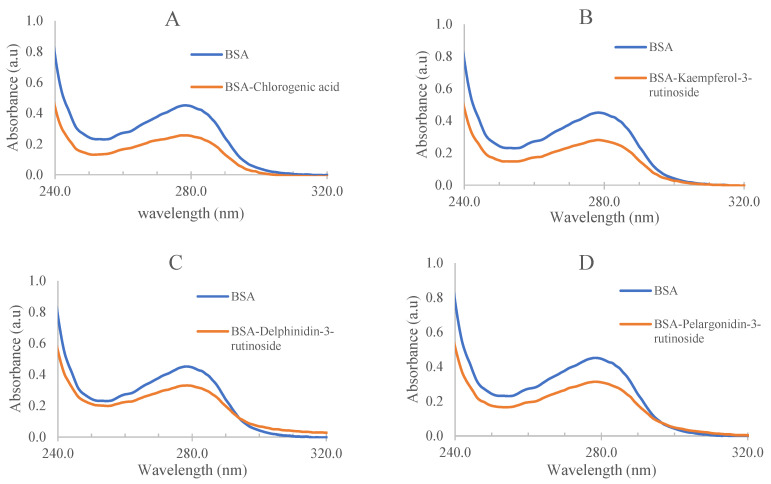
UV-visible absorption spectra of BSA in the absence and presence of (**A**) chlorogenic acid, (**B**) kaempferol-3-rutinoside, (**C**) delphinidin-3-rutioside and (**D**) pelargonidin-3-rutinoside. The concentration of BSA, chlorogenic acid, kaempferol-3-rutinoside, delphinidin-3-rutinoside and pelargonidin-3-rutinoside was 1 × 10^−5^ M for each.

UV-visible absorption spectra are commonly used to investigate the change in structure and formation of a protein–polyphenol complex. They can also confirm if the presence of polyphenols has an influence on the structure of BSA and determine the static quenching between BSA and polyphenols [8]. In our study, UV-visible absorption spectroscopy was used to investigate the interactions between four major polyphenols in tamarillo extract, including chlorogenic acid, kaempferol-3-rutinoside, delphinidin-3-rutioside and pelargonidin-3-rutinoside, and BSA, and the results are shown in Figure 1.

The characteristic absorption maximum of BSA was observed at 280 nm due to the presence of aromatic amino acids including tryptophan, tyrosine and phenylalanine [8]. Absorbances for the four studied polyphenols were observed between 240 nm and 310 nm. Due to the reactions between BSA and polyphenols and the formation of BSA–polyphenol complexes, the maximum absorbance of BSA was significantly (*p* < 0.05) decreased in the presence of polyphenols for all four polyphenols examined. This confirms the existence of static quenching in the presence of polyphenols in BSA. Similarly, other studies have also reported that the binding affinity of BSA to phenols is weakened [11,12].

#### 3.1.2. Fluorescence Spectroscopy

**Figure 2:**
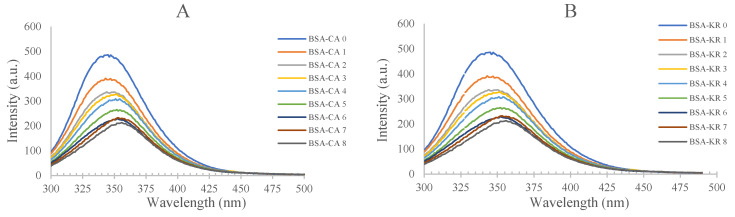

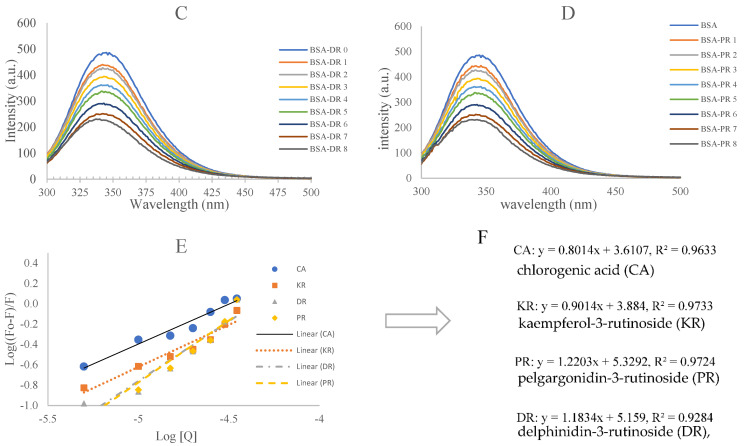
Fluorescence emission spectra of BSA between 300 and 500 nm in the presence of different concentrations of (**A**) chlorogenic acid (CA), (**B**) kaempferol-3-rutinoside (KR), (**C**) delphinidin-3-rutinoside (DR) and (**D**) pelgargonidin-3-rutinoside (PR), and the logarithmic curve for the fluorescence quenching of BSA with CA, KR, DR and PR (**E**). The concentrations of 0 to 8 were 0, 1, 2, 3, 4, 5, 6, 7 and 8 × 10^−5^ M, respectively. (**F**) details the equations of each polyphenol arising from the line of best fit in (**E**) with r^2^ values.

Fluorescence spectroscopy is commonly used to investigate the static quenching of the protein–ligand complex [13]. A decrease in fluorescence was expected to occur in fluorescence quenching, which could have been facilitated by an excited state reaction, ground state complex formation or energy transfer [14]. Dynamic quenching and static quenching are the commonly used quenching mechanisms for fluorescence. Static quenching is caused by a ground state complex formation, which is initiated from a non-fluorescence compound, while dynamic quenching is caused by the colloidal fluorescence substance and quencher [15]. As shown in Figure 2, the excitation wavelength of BSA was found at 280 nm in the fluorescence spectroscopy, and the fluorescence emission peak represented tryptophan, tyrosine and phenylalanine [16]. A downward trend in the fluorescence intensity of BSA was observed with an increasing concentration of polyphenols. These significant decreases and significant shifts in maximum absorption were observed in chlorogenic acid, kaempferol-3-rutinoside, delphinidin-3-rutinoside and pelgargonidin-3-rutinoside from 346 to 358 nm, 346 to 356 nm, 346 to 341 nm and 346 to 343 nm, respectively. These results support the UV-vis spectrophotometer results in Section 3.1.1, which indicated that these four polyphenols interacted with BSA. The fluorescence intensities of BSA with chlorogenic acid, kaempferol-3-rutinoside, delphinidin-3-rutinoside and pelargonidin-3-rutinoside have decreased from 481 to 211, 213, 227 and 231 a.u., respectively. From the results, it was evident that the binding affinity of pelargonidin-3-rutinoside was greater than the other three polyphenols tested. In fact, the structure of polyphenols is highly correlated in the quenching mechanism with BSA, especially in terms of molecular size and the number of hydroxyl groups attached to the benzoic ring. Intermolecular hydrogen bonds and electrostatic interactions are the main driving forces in the formation of BSA-polyphenol complexes [8].

Because the intrinsic fluorescence of BSA was changed after quenching with polyphenols, a set of quenching mechanisms was used to explore the binding parameters and identify the binding site between BSA and polyphenols. The binding parameters were examined using the static quenching mechanism.

When ligand molecules in polyphenols bind independently to a set of equivalent sites on BSA, the number of binding constants and binding sites can be calculated using the equation below [17]:

$$lg\frac{\left(F0-F\right)}{F}=lgKa+nlg[Q]$$

where Ka represents the binding constant of polyphenols with BSA and *n* refers to the number of binding sites for BSA molecule. F0 and F represent the absorption intensity in the absence and presence of quencher in the BSA-polyphenol complex, respectively. [Q] refers to the concentration of quencher in the complex. Therefore, Ka and *n* can be calculated with the equation using the slope and the intercept of a logarithmic curve, respectively, which is shown in Figure 2E. The calculated Ka between chlorogenic acid, kaempferol-3-rutinoside, delphinidin-3-rutinoside and pelargonidin-3-rutinoside and BSA were $6.3\times 10^{6},8.0\times 10^{6},\;1.3\times 10^{7}$ and $1.5\times 10^{7}\;M^{-1}$, respectively (Table 1). Similar results were reported in a previous study on BSA–chlorogenic acid binding at different temperatures [12]. For the remaining tamarillo flavonoids and anthocyanins, their Ka values were very high in comparison with previous findings of plant polyphenol–BSA interactions [18,19]. This difference may be related to the binding sites between polyphenols and BSA. From our results, the values of *n* were in a range of 3.6 to 5.1 (Table 1). As they were greater than 1.0, it was interpreted as the presence of multiple binding sites in the BSA molecule for the polyphenols to bind to. The plots for polyphenols and BSA were linear, which indicates that there was only one type of quenching observed. Delphindin-3-rutinoside had the highest binding affinity to BSA. According to the molecular docking of BSA, the number of cavities on the surface area of BSA indicates the potential binding sites for BSA with polyphenols [20]. This phenomenon was confirmed by [21], who found that all polyphenols interacted with BSA. Meanwhile, they also concluded that at least one binding site on BSA would be involved in the complex interaction near tryptophan residues.

#### 3.1.3. Synchronous Fluorescence Spectroscopy

**Figure 3:**
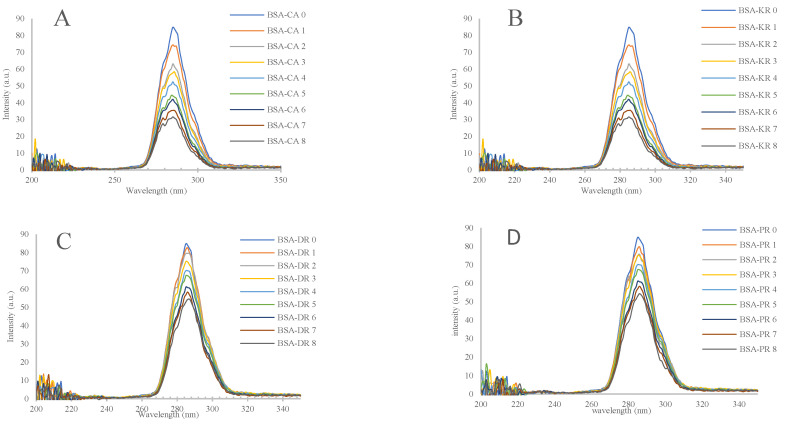

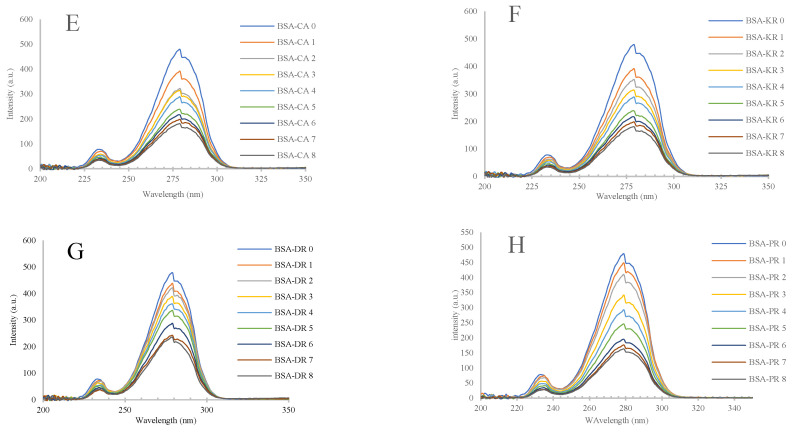
Synchronous fluorescence spectra of BSA in the presence of different concentrations of (**A**) chlorogenic acid (CA), (**B**) kaempferol-3-rutinoside (KR), (**C**) delphinidin-3-rutinoside (DR), (**D**) pelgargonidin-3-rutinoside (PR) at Δλ = 15 nm. Synchronous fluorescence spectra of BSA in the presence of different concentrations of (**E**) chlorogenic acid (CA), (**F**) kaempferol-3-rutinoside (KR), (**G**) delphinidin-3-rutinoside (DR), (**H**) pelgargonidin-3-rutinoside (PR) at Δλ = 60 nm. The concentrations from 0 to 8 were 0, 1, 2, 3, 4, 5, 6, 7 and 8 × 10^−5^ M respectively.

BSA with added polyphenols were analyzed using synchronous fluorescence spectroscopy, which determines the fluorescence peaks in the mixtures based on the conformational changes and the interactions between BSA and polyphenols. Since most of the interactions related to BSA have been reported to be found in the vicinity of aromatic amino acids, tryptophan and tyrosine residues [8,20,21], the Δλ was set to 15 and 60 nm between the excitation and emission wavelength to target for the conformational change in these two residues, respectively [22]. The change in maximum wavelength was expected to explain the change in conformation of tryptophan and tyrosine. As shown in Figure 3A–D, the intensities of fluorescence on tryptophan decreased with increasing concentrations of polyphenols. The conformational change in the tryptophan residue was observed by increasing accessibility to hydrophilic group of polyphenols [8]. Moreover, similar results were observed for the tyrosine residue (Figure 3E–H). The conformational change in the tyrosine residue on BSA may be caused by the increasing probability of exposure to a hydrophilic environment in the presence of polyphenols. Regarding the quenching of fluorophore in the binding mechanism, the interaction between BSA and polyphenols from tamarillo was located in the tryptophan and tyrosine residues of BSA.

### 3.2. Physicochemical Properties of Fresh Milk Cream Fortified with Polyphenols from Tamarillo Extract

#### 3.2.1. Particle Size Distribution

**Figure 4:**
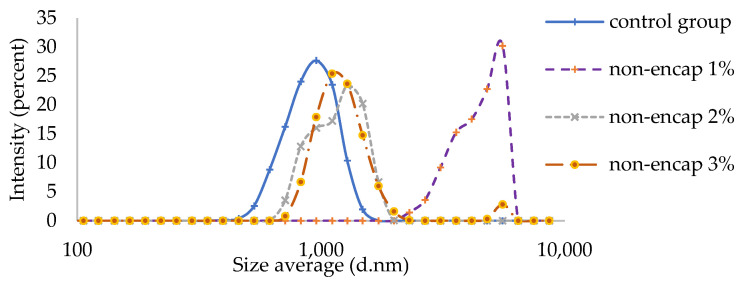
Particle size distribution of cream samples mixed with different concentrations of tamarillo extracts without encapsulation at 4 °C (*n* = 3). The control group refers to cream.

The particle size distribution of the control group ranged from 396 to 1720 nm with an average *D* _[3, 2]_ of 1688.84 ± 333 nm, as shown in Figure 4. The particle size distribution of the cream samples with the addition of 2% extract without encapsulation ranged from 712 to 1990 nm, with an average *D* _[3, 2]_ of 3363 ± 886 nm (Table 2). A similar particle size range for the cream samples was observed with the addition of 3% extract without encapsulation. Interestingly, the widest range of particle size distribution in the cream samples was observed with the addition of 1% tamarillo extract from 2300 nm to 5560 nm with an average *D* _[3, 2]_ of 4419 ± 1205 nm. In general, the average droplet size of our creams (with and without tamarillo extract) was similar to the findings in previous studies, which reported that the particle size distribution of individual fat globules in cream ranged from 300 to 9000 nm [23,24]. The average size distribution of the cream samples significantly increased with the addition of 1% tamarillo extract (Figure 4). This phenomenon is likely to be associated with the BSA–polyphenol interactions.

As discussed in Section 3.1.2, the binding affinity of tamarillo polyphenols to BSA indicated that more than one binding site of tryptophan residues in BSA was involved in protein–polyphenol interactions. Because milk proteins (including BSA) are strong emulsifiers for oil-in-water (O/W) emulsions like cream, the aggregation of milk protein caused by the linkages with tamarillo polyphenol can lead to coalescence between fat globules, which in turn results in an increase in average droplet size. However, when the concentration of tamarillo polyphenols was over 1%, the average droplet size decreased when non-encapsulated (Figure 4). In fact, the aggregation of the protein–polyphenol complex and the coalescence between fat globules were also affected by the electrostatic reaction and the presence of hydrogen bonds between polyphenols and the aqueous phase [25]. We, therefore, conclude that if the concentration of polyphenols is greater than 1%, some steric hindrance effects may occur to prevent the coalescence of oil droplets, resulting in a smaller average droplet size in cream.

#### 3.2.2. Measurement of Rheological Properties

**Figure 5:**
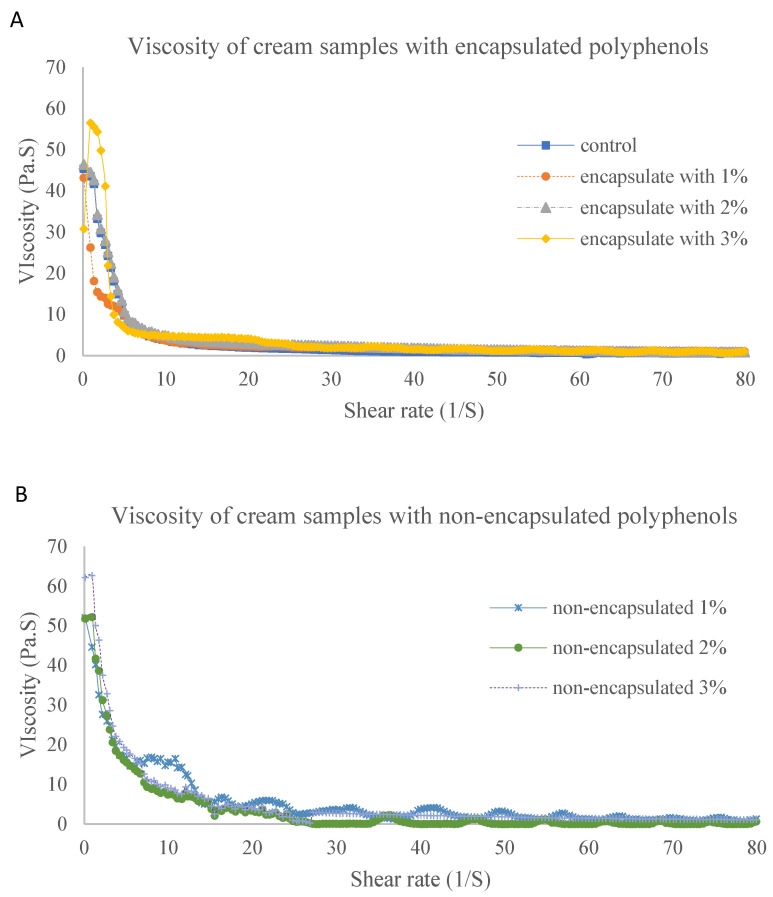
Viscosity of cream samples with (**A**) and without (**B**) encapsulated tamarillo polyphenols. The amount of tamarillo extract added was 1%, 2% and 3% (*w*/*w*) of the cream weight.

According to Figure 5, the rheological property of the cream was shear-thinning. A similarity in rheological properties of the cream mixture was observed between the control group, cream with encapsulated polyphenols and cream with non-encapsulated polyphenols. This can be explained by the presence of maltodextrin in the encapsulation process of polyphenols. Encapsulation stability was improved with the addition of maltodextrins against the oxidation of chemicals. The viscosity of the cream was quite stable at a high shear rate from 20 to 60 (1/s), regardless of the concentration of added polyphenols. The addition of encapsulated extracts did not influence the rheological properties of the cream, with the protection of polyphenols inside the amorphous carbohydrate microstructure matrices. The particle size may also have influenced the rheological properties of the cream. As shown in Figure 4 and Table 2, the particle size distribution of the cream samples fortified with 1% of extracted polyphenols was the widest with the lowest fluidity among all samples. As the enlarged particle size was caused by the formation of coalescence between cream proteins and polyphenols, higher energy was required to break the intermolecular forces. Thus, the lowest fluidity of samples was observed with the addition of 1% polyphenols. In other words, with increasing particle size, the coalescence between oil droplets decreases, and the fluidity of the cream samples increases.

#### 3.2.3. Phenolic and Anthocyanin Content

According to Table 3, there was a significant (*p* < 0.05) difference in the phenolic and anthocyanin contents between the cream samples with added tamarillo extract at 1, 2 and 3% (*w*/*w*). A two-way ANOVA was used to compare the samples in each group with the null hypothesis that the mean between each group was equal and that they came from the same population mean. The concentration of chlorogenic acid was 14.977 μg.mL^−1^ in the control group, which is equivalent to 33.28 mg chlorogenic acid in 100 g of dry weight (DW) of the samples. This was much lower than the previous findings of our research group [1], which was 66.35 ± 1.1 mg.100 g^−1^ DW of samples. However, the amount of chlorogenic acid present in the tamarillo extract was significantly higher than that in the non-encapsulated tamarillo extracts in the cream samples by 11.07- to 19.78-fold for the addition of 1 to 3% tamarillo extract, respectively. Chlorogenic acid in the cream sample with the addition of 3% non-encapsulated extract was 1.353 μg.mL^−1^, although we were expecting it to be around 2.2 μg.mL^−1^. The decreased amount of chlorogenic acid in the cream samples may have resulted from its interactions with milk proteins, including BSA.

For kaempferol-3-rutinoside, the concentration in the control group was 7.722 μg.mL^−1^, which is equivalent to 17.16 mg.100 g^−1^ DW of the samples. The concentrations of non-encapsulated kaempferol-3-rutionside in the cream samples were significantly higher than that in the encapsulated kaempferol-3-rutionside. With increasing the concentration of polyphenol extracts from 1% to 3%, the concentration of kaempferol-3-rutinoside in the cream mixture was also increased in both groups. The amount of kaempferol-3-rutinoside in natural tamarillo was significantly higher than that in the non-encapsulated tamarillo in the cream mixture by a range from 13.62- to 41.52-fold. Similar results were observed for chlorogenic acid due to the interactions between BSA and polyphenols; i.e., the sample with the addition of 3% non-encapsulated extract had a lower concentration of kaempferol-3-rutinoside than that of the expected value of 0.558 μg.mL^−1^. In contrast to chlorogenic acid, the extensive differences in kaempferol-3-rutionside between the natural tamarillo and non-encapsulated extracts in the cream samples demonstrated that there would be higher binding affinity toward BSA with kaempferol-3-rutinoside than with chlorogenic acid. Hydrogen bonds and electrostatic interaction are the main driving forces related to the binding of BSA with polyphenols [25]. The increased glycosylation of chlorogenic acid may have decreased the binding affinity with BSA if the binding site was located in domain IIA of BSA [20].

For delphidinin-3-rutinoside, the concentration without encapsulation was 81.165 μg.mL^−1^, which is equivalent to 180.37 mg.100 g^−1^ DW. This was much lower than the results of [1], who reported a concentration of 254.76 ± 6.33 mg.100 g^−1^ DW, although the same type of tamarillos was used with an increase in the addition of extracted polyphenols in cream from 1% to 3%.

The concentration of delphidinin-3-rutinoside was higher in the cream samples when the polyphenols were not encapsulated compared to that when they were encapsulated. A 62.5 times, 220 times and 377 times higher concentration of delphidinin-3-rutinoside was found in 1%, 2% and 3% fortification, respectively. The delphinidin-3-rutinoside present in the original tamarillo was significantly higher than the values in the cream mixture by a range between 7.2- and 18.4-folds. In comparison with the cream samples in the presence of chlorogenic acid, a similar range of differences were observed between the natural tamarillo extracts and non-encapsulated extracts in the cream for delphinidin-3-rutinoside. This may be explained by delphidinin-3-rutinoside having similar number of glycosyl groups as chlorogenic acid, which contributes to a similar binding affinity with BSA.

Interestingly, in the encapsulated groups with maltodextrin, the concentration of polyphenols decreased with an increase in the addition of tamarillo extracts. For pelargonidin-3-rutinoside, 10.953 μg.mL^−1^ was found in the control, which is equivalent to 24.34 mg.100 g^−1^ DW of the sample. Again, the concentration of pelargonidin-3-rutinoside was 8 times higher in our previous study [1] than the current result. The concentrations in the non-encapsulated group were directly increased with the increase in the addition of polyphenol extracts. A similar decreasing trend in concentration was obtained in the cream samples with the presence of maltodextrin. In contrast, the concentration differences in pelargonidin-3-rutinoside between natural polyphenols and non-encapsulated polyphenols with the cream were lower than that of delphnidin-3-rutinoside by 9.4- to 26-fold, respectively. This means that the ability of BSA to bind with pelargonidin-3-rutinoside falls between chlorogenic acid and delphinidin-3-rutinoside, whilst kaempferol-3-rutinoside has the highest binding affinity with BSA.

According to these differences between encapsulated and non-encapsulated tamarillo extracts when added to cream, the amounts of overall polyphenols presented in the cream samples were significantly lower in the encapsulated polyphenols. This result might be explained by an insufficient concentration of coating agent during encapsulation. Maltodextrin was supposed to improve the antioxidant activity of polyphenols as it sustains good solubility in solid content. Normally, the microencapsulation efficiency of nano-nutrients with maltodextrin is obtained with a maximum value of 82% with 60% maltodextrin in total weight of samples [26]. Several studies have reported that the encapsulation efficiency of polyphenols from saffron extracts is enhanced up to 90% in recovery with the combination of more than 37.6% of maltodextrin, 2% of gum arabic and 0.4% gelatin or other whey protein concentrates [27,28]. Ref. [27] also reported that the total solid content of these coating agents positively influenced the emulsion viscosity by reducing the formation time of crust on the surface of droplets. This conclusion supports our results shown in Figure 5.

The results from the two-way ANOVA indicated that encapsulation significantly influenced the polyphenol content in the cream samples, whilst the percentage of polyphenol extract added did not significantly interact with encapsulation on the polyphenol content. Furthermore, the concentration of polyphenol extract was not significantly affected by the phenolics present in the cream samples.

#### 3.2.4. Total Phenolic Content (TPC) and Antioxidant Capacity

**Figure 6:**
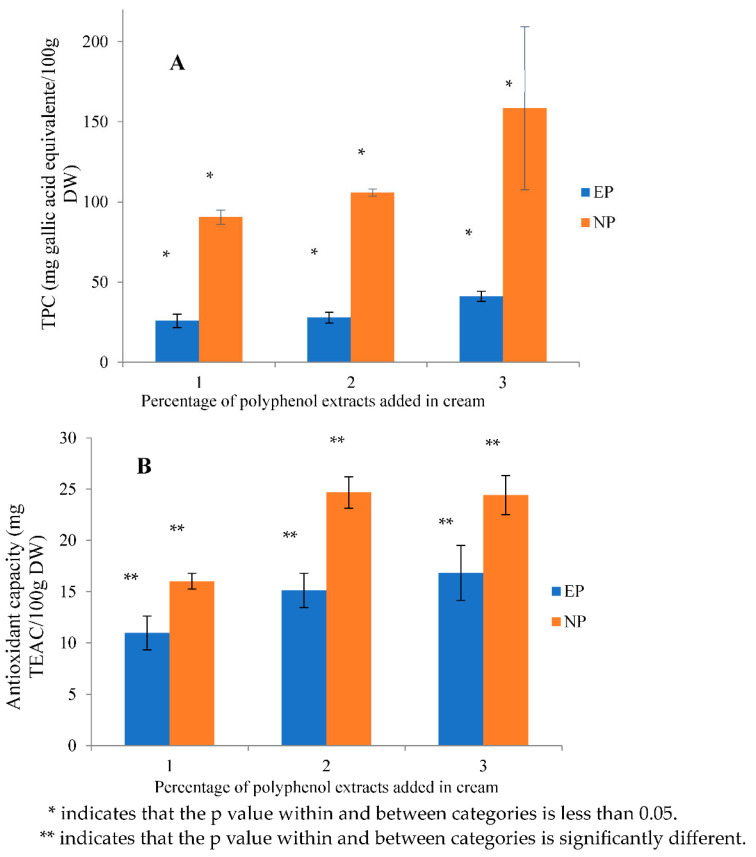
(**A**) The TPC and (**B**) antioxidant capacity of the cream samples with the addition of encapsulated (EP) and non-encapsulated (NP) polyphenol extracts at concentrations of 1, 2 and 3% (*w*/*w*) (*n* = 3). Standard curves used to calculate the data can be found in the data file in the Supplementary Materials and Figures S3 and S4 for gallic acid and TEAC, respectively.

The TPC was 599.69 mg GAE.100 g^−1^ of dried samples from the original tamarillo, which was close to the previously reported value of 707.04 ± 30.65 mg GAE.100 g^−1^ DW [1]. From Figure 6A, TPC increased with increasing the concentration of extracts from 1% to 3% in both groups. The TPC in the non-encapsulated groups was significantly higher than that in the cream samples with encapsulated polyphenols by 3.51, 3.80 and 3.86 times for 1, 2 and 3% added polyphenols, respectively. Moreover, the differences between non-encapsulated groups sharply increased with the increase in polyphenol concentration, whilst a slightly increasing trend was observed in the encapsulated group. The recovery of the TPC of tamarillo extracts in the cream samples ranged from 25.93% to 28.49% after encapsulation. This shows that the interaction between polyphenols and cream proteins still occurred when encapsulated with maltodextrin. The results are supported by another study [29], which found that the TPC of polyphenol extracts was significantly decreased after encapsulation with 30% maltodextrin. They only observed 42.8% recovery efficiency from encapsulation. As for this study, we only utilized 10% maltodextrin, which may have been insufficient to provide practical evidence of the successful encapsulation of tamarillo extracts.

The antioxidant activity was measured using the CUPRAC method. The control group without the addition of tamarillo extract showed 453.54 mg TEAC.100 g^−1^ DW, which is equivalent to 18.11 μM TEAC.g^−1^ of the dried samples. The antioxidant capacity increased with increasing concentration of tamarillo extract for both groups (Figure 6B). The antioxidant capacity in the non-encapsulated groups was relatively higher than that in the cream samples in the encapsulated groups. In comparison with the non-encapsulated groups, the antioxidant capacity in the encapsulated group was found to be 0.68, 0.61 and 0.69 times lower with 1, 2 and 3% of polyphenol extract, respectively. With the increasing addition of polyphenol extract, the differences in antioxidant capacity became smaller in both groups. The recovery of antioxidant capacity in the cream samples was increased from 61% to 69% with 1% to 3% polyphenols. It can be concluded that the functionality of bioactive compounds would be retained with the encapsulation using maltodextrin. As discussed above, the lower antioxidant capacity of polyphenols with 10% maltodextrin alone as the encapsulating agent might be caused by the low ability to stabilize the microcapsule around the polyphenol extracts and the low ability to prevent polyphenols from interacting with milk proteins and oxygen. Antioxidant capacity is significantly correlated to the TPC. To improve the preservation of bioactive compounds from tamarillo, the ratio of extracted polyphenols to the coating agent mixture (which combines maltodextrin and gum arabic (50% *w*/*w*)) should be increased from 10% to 30% [30]. Statistical analysis using a two-way ANOVA indicated that encapsulation significantly influenced the TPC and antioxidant capacity, but the concentration of polyphenol extracts did not significantly interact with encapsulation on the functionality of bioactive compounds.

As a result, the chromogenic redox reagent used in the CUPRAC method was stable, capable and easily handled to analyze antioxidants from tamarillo. A complicated procedure to exhaust oxygen during the extraction of polyphenols was not required as the oxidation reaction with the chromogenic redox used in CUPRAC was faster than with oxygen [31]. However, the limitation of this experiment related to the use of Trolox should be considered as the oxidation would occur more rapidly in the presence of oxygen than in polyphenols. The reagent needed to be kept at −80 °C for 4 months before use [3]. Another aspect of decreased bioactive compounds in encapsulated polyphenols added to cream samples is dependent on the water-binding affinity of maltodextrin. The low molecular weight of maltodextrin was found to have high water-binding capacity and high water solubility in the formation of protein–maltodextrin complex particles by covalent bonds after encapsulation [27,32].

## 4. Conclusions

The interaction mechanism between BSA and polyphenols was examined using UV spectroscopy, fluorescence and synchronous spectroscopy. Fluorescence results indicated that static quenching was the main mechanism in the fluorescence spectra, and tryptophan and tyrosine residues were the two major aromatic amino acids that were related to BSA interactions. Results from synchronous fluorescence spectroscopy indicated that there were more than three binding sites for the selected polyphenols to BSA, and there were at least three binding sites near the tryptophan residue on BSA. This may cause a lower binding affinity of BSA to polyphenols. The results for physicochemical properties showed that the interaction between BSA and polyphenol extracts from tamarillo had a light effect on the rheological property of the cream. The concentration of bioactive compounds decreased when interacted with cream protein. The higher the concentration of polyphenol extracts added to the cream, the higher the bioactivities that were measured. With the application of encapsulation with 10% of maltodextrin on polyphenol extracts, the bioactive compounds measured in the cream mixture were significantly decreased, which may be caused by an insufficient concentration of maltodextrin, water-binding affinity and the binding affinity of maltodextrin to cream protein. Therefore, the use of 10% maltodextrin as an encapsulation agent on polyphenol extracts alone may not be suitable to protect bioactive compounds in the form of microcapsules in cream. In future research, this should be improved with the use of other polysaccharides or lipids.

## Figures and Tables

**Table 1:** Binding sites and binding constants (Ka) for fluorescence spectra.

BSA Mixture	CA	KR	PR	DR
*n* (binding sites)	3.61	4.06	3.86	5.10
lgKa	0.80	0.90	1.12	1.18
Ka (10^6^ $M^{-1}$)	6.33	7.97	13.24	15.25

**Table 2:** Particle size distribution profile of the cream samples with different concentrations of tamarillo extracts without encapsulation (*n* = 3). Data are presented as mean ± standard deviation. Different letters indicate statistical differences (*p* < 0.05) between groups.

	% Polyphenols	Z Average (d. nm)	*D* _[3, 2]_ (nm)
Control group	0	1689 ^a^ ± 333	1688.84 ^a^ ± 332.87
Non encapsulated	1	4418 ^b^ ± 1204	4418.67 ^b^ ± 1204.77
	2	3362 ^c^ ± 886	3362.89 ^c^ ± 886.14
	3	1970 ^d^ ± 191	1969.63 ^d^ ± 191.16

**Table 3:** Phenolic and anthocyanin contents in the cream fortified with tamarillo extracts, either with or without encapsulation (*n* = 3). Data are presented as mean ± standard deviation. Different letters indicate statistical differences (*p* < 0.05) between groups.

Concentration of Polyphenols (μg/mL)		% Polyphenols Added						
		Control Group	Non-Encap 1%	Non-Encap 2%	Non-Encap 3%	Encap 1%	Encap 2%	Encap 3%
Phenolics	Chlorogenic acid	14.977 ^a^ ± 0.976	0.757 ^b^ ± 0.321	1.057 ^c^ ± 0.012	1.353 ^d^ ± 0.063	0.263 ^e^ ± 0.009	0.375 ^f^ ± 0.035	0.484 ^g^ ± 0.035
	Kaempferol-3-rutinoside	7.722 ^a^ ± 0.431	0.186 ^b^ ± 0.008	0.399 ^c^ ± 0.012	0.567 ^d^ ± 0.017	0.079 ^e^ ± 0.006	0.175 ^f^ ± 0.004	0.238 ^g^ ± 0.022
Anthocyanin	Delphinidin-3-rutinoside	81.165 ^a^ ± 2.950	4.403 ^b^ ± 0.223	8.611 ^c^ ± 0.491	11.283 ^d^ ± 0.540	0.064 ^e^ ± 0.006	0.039 ^f^ ± 0.0003	0.030 ^g^ ± 0.005
	Pelargonidin-3-rutinoside	10.953 ^a^ ± 0.634	0.421 ^b^ ± 0.020	0.861 ^c^ ± 0.040	1.163 ^d^ ± 0.047	0.013 ^e^ ± 0.003	0.009 ^f^ ± 0.0007	0.007 ^g^ ± 0.0007

Note: calibration curves can be found in the Supplementary Materials Figure S2A–D. Non-encap indicates non-encapsulated polyphenols. Encap indicates encapsulated polyphenols.

## Data Availability

All data are contained within the article and Supplementary Material.

## Supplementary Materials

The following supporting information can be downloaded at: https://www.mdpi.com/article/10.3390/antiox12081611/s1, Figure S1. A photograph showing the encapsulated tamarillo extract with maltodextrin in 10% *w*/*w*. Figure S2. Calibration curve of phenolic and anthocyanin content determined using LC-MS; Figure S3. Standard curve of total phenolic content measurement with Folic–Ciocalteu reagent; Figure S4. Standard curve of antioxidant capacity measured using the4 CUPRAC method.
