# Supplementary material for: Interaction between Bovine Serum Albumin in Fresh Milk Cream and Encapsulated and Non-Encapsulated Polyphenols of Tamarillo
Source: Antioxidants (Basel). 2023 Aug 14;12(8):1611. doi: 10.3390/antiox12081611 (PMC10451476; doi:10.3390/antiox12081611)
Supplement: Supplementary file 1 [file antioxidants-12-01611-s001.zip › antioxidants-2498466-supplementary.pdf]

Supplementary Materials:

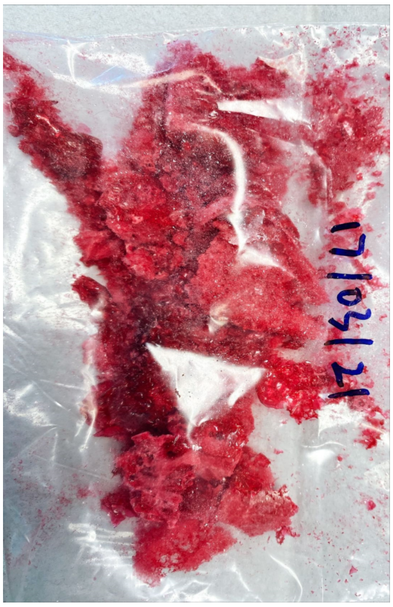

Figure S1. A photograph of the encapsulated tamarillo extract with maltodextrin, in 10% w/w.

Calculation of total phenolic content in mg of gallic acid equivalent per g of extracted powder:

$$= \frac{\text{Final concentration (mg/L) x volume of extract (0.01L) x dilution factor (100)}}{weight\ of\ sample\ (g)}$$

Calculation of antioxidant capacity in mg of Trolox equivalent per g of extracted powder:

$$= \frac{Final\ concentration\ \left(\frac{mg}{L}\right) \times volume\ of\ extract\ (0.01L)}{weight\ of\ sample\ (g)}$$

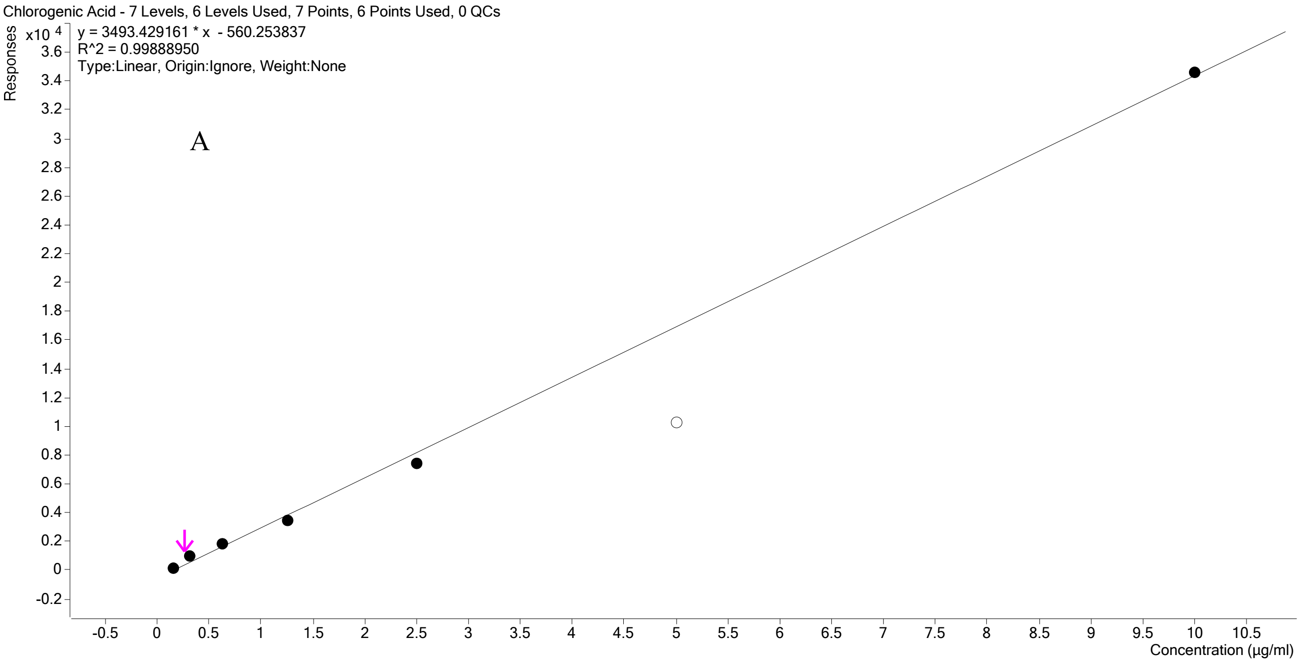

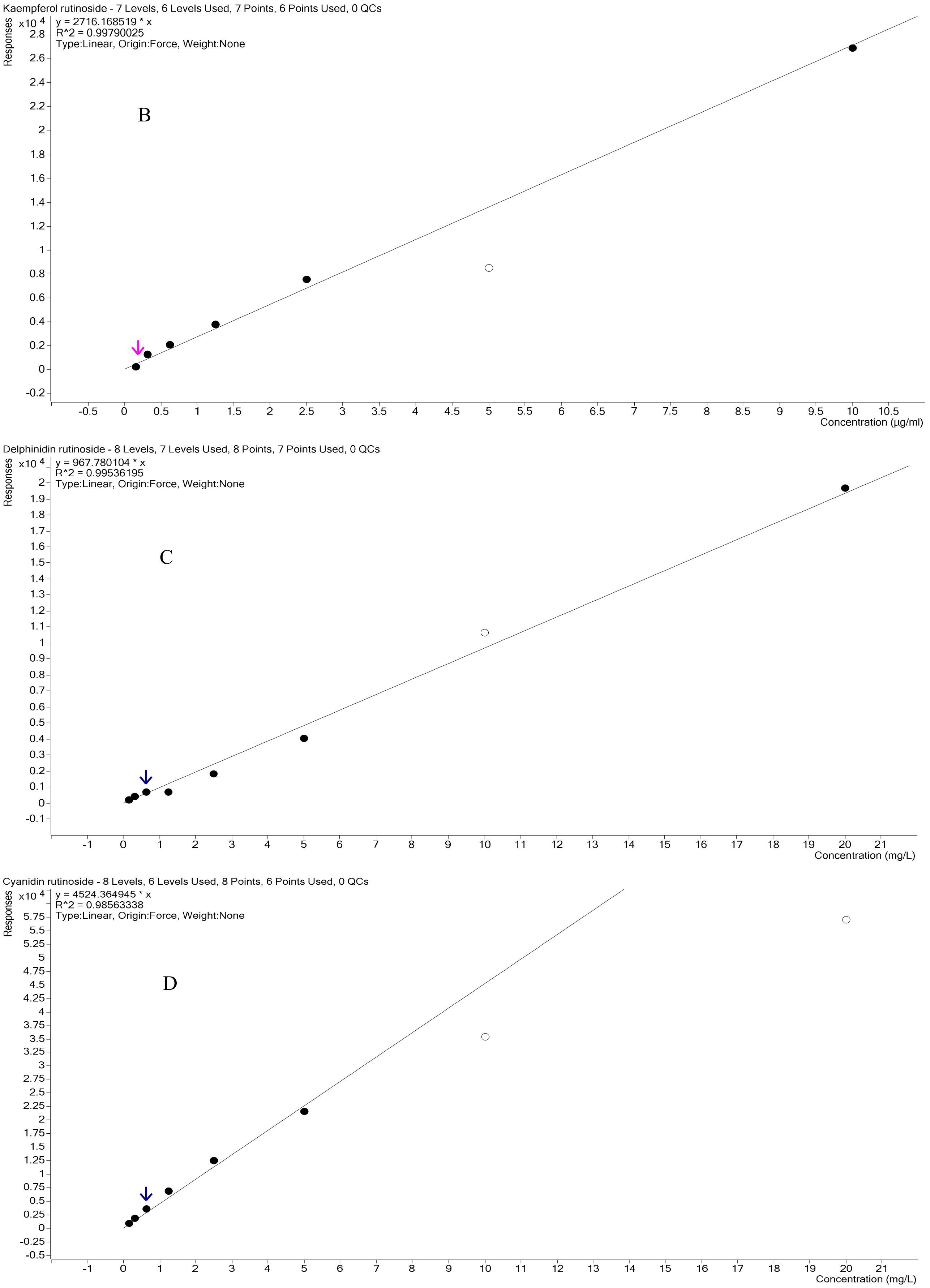

Figure S2. Calibration curve of phenolic and anthocyanin content determined by LC-MS. A represents the concentration of chlorogenic acid in unit of μg/L, B represents the concentration of kaempferol-3-rutinoside in

μg/L, C represents the concentration of delphinidin-3-rutinoside in mg/L, and D represents the concentration of pelargonidin-3-rutinoside in mg/L.

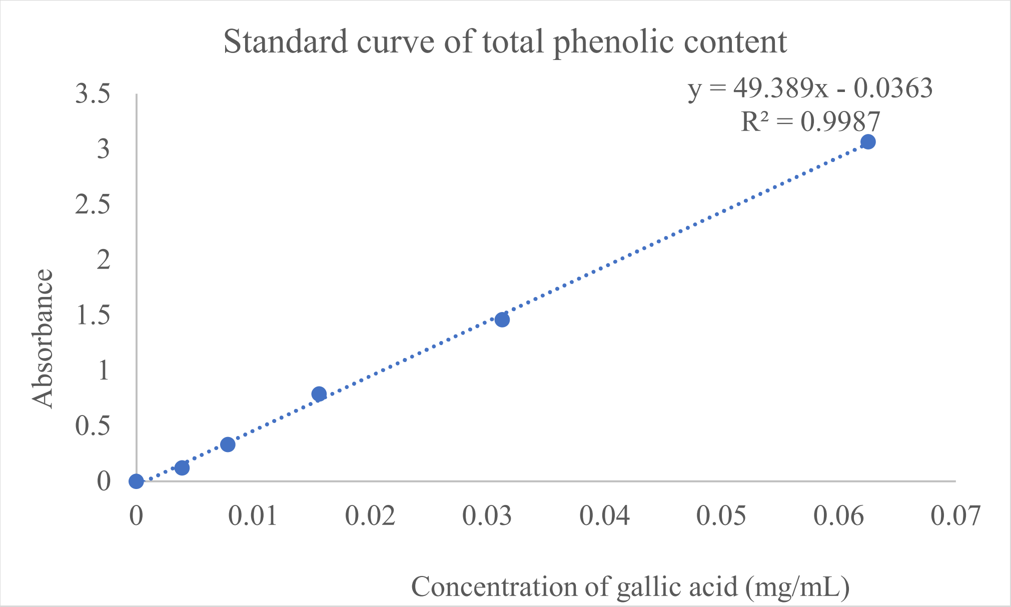

Figure S3. Standard curve of total phenolic content measurement with Folic Ciocalteu reagent.

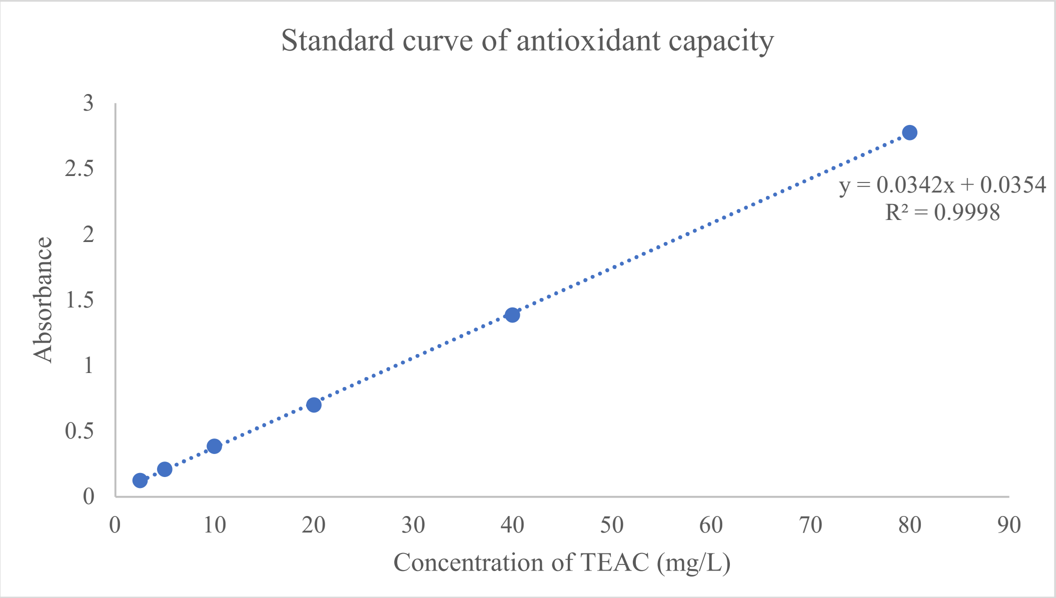

Figure S4. Standard curve of antioxidant capacity measured by CUPRAC method.
